# Altered Trabecular Bone Structure and Delayed Cartilage Degeneration in the Knees of Collagen VI Null Mice

**DOI:** 10.1371/journal.pone.0033397

**Published:** 2012-03-20

**Authors:** Susan E. Christensen, Jeffrey M. Coles, Nicole A. Zelenski, Bridgette D. Furman, Holly A. Leddy, Stefan Zauscher, Paolo Bonaldo, Farshid Guilak

**Affiliations:** 1 Department of Orthopaedic Surgery, Duke University Medical Center, Durham, North Carolina, United States of America; 2 Department of Biomedical Engineering, Duke University, Durham, North Carolina, United States of America; 3 Department of Mechanical Engineering & Materials Science, Duke University, Durham, North Carolina, United States of America; 4 Department of Histology, Microbiology and Medical Biotechnologies, University of Padova, Padova, Italy; University of Rochester, United States of America

## Abstract

Mutation or loss of collagen VI has been linked to a variety of musculoskeletal abnormalities, particularly muscular dystrophies, tissue ossification and/or fibrosis, and hip osteoarthritis. However, the role of collagen VI in bone and cartilage structure and function in the knee is unknown. In this study, we examined the role of collagen VI in the morphology and physical properties of bone and cartilage in the knee joint of *Col6a1^−/−^* mice by micro-computed tomography (microCT), histology, atomic force microscopy (AFM), and scanning microphotolysis (SCAMP). *Col6a1^−/−^* mice showed significant differences in trabecular bone structure, with lower bone volume, connectivity density, trabecular number, and trabecular thickness but higher structure model index and trabecular separation compared to *Col6a1^+/+^* mice. Subchondral bone thickness and mineral content increased significantly with age in *Col6a1^+/+^* mice, but not in *Col6a1^−/−^* mice. *Col6a1^−/−^* mice had lower cartilage degradation scores, but developed early, severe osteophytes compared to *Col6a1^+/+^*mice. In both groups, cartilage roughness increased with age, but neither the frictional coefficient nor compressive modulus of the cartilage changed with age or genotype, as measured by AFM. Cartilage diffusivity, measured via SCAMP, varied minimally with age or genotype. The absence of type VI collagen has profound effects on knee joint structure and morphometry, yet minimal influences on the physical properties of the cartilage. Together with previous studies showing accelerated hip osteoarthritis in *Col6a1^−/−^* mice, these findings suggest different roles for collagen VI at different sites in the body, consistent with clinical data.

## Introduction

Collagen VI is heterotrimeric protein consisting of three different α-chains: α1(VI), α2(VI), and α3(VI). Collagen VI has been shown to play a bridging role in connective tissues, where it forms a flexible network interlinking collagen types I, II, IV, proteoglycans, hyaluronan, and cells [1], [2], [3], [4], [5]. Recent research has identified three other collagen VI subunits, α4(VI), α5(VI), and α6(VI), which may substitute in place of α3(VI) in the heterotrimeric fibrils [6], [7]. Human collagen VI genetic abnormalities have been linked to muscular disorders, specifically Bethlem myopathy, Ullrich congenital muscular dystrophy and congenital myosclerosis [8], [9], [10], [11], and to ligamentous disorders, namely ossification of the ligamentum flavum, ossification of the posterior longitudinal ligament of the spine, and diffuse idiopathic skeletal hyperostosis [12], [13], [14]. Mutations in collagen VI genes have also been linked to mitochondrial dysfunction [15], to abnormal expression of proteoglycans and adhesion molecules in some tissues [16], [17], and to a skin disorder, atopic dermatitis [7]. Recently, a susceptibility locus for atopic dermatitis was identified in *COL6A5* [18] and the *DVWA* (double von Willebrand factor A domains) susceptibility locus for knee osteoarthritis identified as part of *COL6A4* [19], [20].

In addition to its important role in muscle, collagen VI is present in most other musculoskeletal tissues. In articular cartilage, collagen VI is normally found distinctly localized to the pericellular matrix (PCM) [21] in conjunction with other molecules such as collagen IX, decorin, fibronectin, and hyaluronan, where it is believed to help anchor the chondrocyte to the PCM [1], [22], [23]. In this respect, it is hypothesized that collagen VI is critical for proper transduction of biochemical and biomechanical signals to the cell [24], [25]. In bone, collagen VI has been identified both in remodeling bone [26] and in developing bone, where it may play a role in bone formation at the growth plate [27], with evidence for its role in regulating collagen I expression in the early phase of IL-4-induced mineralization [28]. Collagen VI has also been found in other tissues of the knee, including the synovium [29], electron-dense seams of ligaments [30], and the cellular, pericellular, and main-body regions of the menisci [31], [32], as well as in other cartilaginous tissues such as the intervertebral disc [33].

In mice, targeted gene disruption of *Col6a1* prevents the formation of collagen VI. Although these mice do not exhibit any major physical abnormalities, the disruption of *Col6a* results in muscular defects characteristic of Bethlem myopathy [15], [34]. *Col6a1* ^−/−^mice also exhibit skeletal abnormalities including delayed development and ossification, as well as accelerated osteoarthritic changes in the hip joint in comparison to *Col6a1^+/+^*controls [27]. However, clinical studies have shown that collagen VI mutations may have significantly differing effects on proximal versus distal joints of the body, depending on the specific mutation [9]. For example, Bethlem myopathy is generally characterized by proximal muscle weakness and distal joint contractures, while Ullrich congenital muscular dystrophy is associated with proximal joint contractures, distal hyperlaxity and global muscle weakness [9]. As muscle weakness and joint laxity can alter joint loading and are associated risk factors for osteoarthritis, the potential influence of collagen VI deficiency on osteoarthritis may vary among different joints of the body.

The goal of this study was to examine the hypothesis that the absence of collagen VI affects the development and age-related changes of bone and cartilage in the knee joint. The knees of *Col6a1^+/+^* and *Col6a1^−/−^* mice were assessed by quantitative micro-computed tomography (microCT) and histologic analysis. The micro-scale physical properties of the articular cartilage were determined using atomic force microscopy (AFM) to measure the elastic modulus, roughness, and frictional coefficient of the articular surface, and scanning microphotolysis (SCAMP) to measure the diffusional transport properties of the cartilage.

## Materials and Methods

### Tissue Preparation

All studies and protocols involving animals were approved by the Duke University Institutional Animal Care and Use Committee (registry number A137-09-05). *Col6a1^+/+^* and *Col6a1^−/−^* mice, created by Bonaldo et al. [34], were raised on a CD1 genetic background [27]. At the target age of 2, 9, or 15 months old (*n* = 8–9 mice per group, 4 males and 4 or 5 females) were sacrificed and frozen at −20°C. The final 15-month time point was chosen with consideration that 80% of C57BL/6J mice survive to 24 months, and spontaneous knee arthritis occurs in 39% at age 12–17 months [35]. The earliest time point was chosen to demonstrate a still-maturing skeleton. Although in some cases, freezing at −20°C has been shown to alter mechanical properties of articular cartilage [36], all samples were treated equally and thus freezing should not affect overall outcome. The left and right legs were isolated and thawed for dissection directly before fixation (right limb: microCT and histology studies) or before microscopy (left limb: AFM and diffusion studies).

### Microcomputed Tomography (MicroCT)

Right knee joints were fixed in 10% buffered neutral formalin. Knees were scanned by a microCT system (microCT 40, Scanco Medical AG, Bassersdorf, Switzerland) using scan acquisition parameters of 55 kV and intensity of 144 µA medium resolution (16 µm) images were produced. A hydroxyapatite (HA) calibration phantom was used to scale values of linear attenuation for the calcified tissues to known density values of 100, 200, 400 and 800 HA mg/cc [37]. All segmentation and morphology measures were performed within the Scanco software. Bone morphology was evaluated along the long axis of the tibia in two regions, the tibial epiphysis proximal to the growth plate and the metaphyseal region in the 25 contiguous slices immediately distal to the fibular attachment, as described previously [38]. In the tibial epiphysis, the cortical and trabecular bone together were evaluated for total volume (TV, mm^3^), bone volume (BV, mm^3^), bone volume fraction (BV/TV), and bone density (BD, mg HA/cm^3^), and the trabecular bone alone was evaluated for structure model index (SMI, a quantitative measure of the shape of the trabeculae, which tends to increase with age and/or disease) [39], [40], connectivity density (ConnDens, 1/mm^3^), trabecular number (Tb.N, 1/mm), trabecular thickness (Tb.Th, mm), and trabecular separation (Tb.Sp, mm). In the tibial metaphysis, bone volume (BV, mm^3^) and bone tissue density (BD, mg HA/cm^3^) were evaluated for both cortical and trabecular bone. These values were multiplied to calculate mineral content (µg). The length of the tibial epiphysis was estimated by counting the number of 16-µm slices from the subchondral bone of the tibial plateau distally to the growth plate; the lengths of two subsets of this region, the trabecular and non-trabecular (subchondral bone) portions, were measured.

### Histology

Following scanning by microCT, intact knee joints were decalcified, dehydrated in EtOH, embedded in paraffin, and sectioned by microtome into 7-µm coronal slices perpendicular to the long axis of the tibial. Sections were stained with hematoxylin, Fast Green, and safranin-O. Digital micrographs were taken of the slides (Olympus BX41 microscope, Olympus DP-11 camera, Olympus, Center Valley, PA).

The subchondral bone thickness was measured across the full width of the loaded regions of the femoral condyle and tibial plateau in histologically stained sections. These subchondral thickness measures did not include calcified cartilage. For each of the medial and lateral condyles and regions of the tibial plateau, ten lines were drawn from the internal edge of the calcified cartilage to the nearest bone marrow. Lines were equally spaced along the subchondral bone and perpendicular to the cartilage surface. These lengths were averaged to determine the mean subchondral bone thickness for the joint. Additionally, the thickness of the joint capsule was measured on the medial side of the joint adjacent to the meniscus. This joint capsule, which primarily included the medial collateral ligament (MCL), was measured with five lines drawn perpendicular to the joint space adjacent to the meniscus. Line lengths were averaged.

Three experienced, blinded graders (*JMC, BDF, HAL*) evaluated histological samples to assess arthritic progression according to a modified Mankin score for mouse knee joints, as described previously [37]. Briefly, articular cartilage structure, tidemark duplication, safranin-O staining, fibrocartilage, hypertrophic chondrocytes and subchondral bone were assessed for a score of 28 points. Four sites were evaluated: the lateral femoral condyle, lateral tibial plateau, medial femoral condyle, and medial tibial plateau. The scores from all graders for each site were averaged and summed to a single score ranging from 0 to 112, with higher scores indicating greater levels of osteoarthritic joint degeneration. Average scores for each experimental group were reported with standard error indicated.

Three blinded graders experienced in knee joint morphology (*BDF, FG, HAL*) graded the same histologic samples to asses osteophyte development according to a grading protocol based on previously reported histomorphological and cell biological parameters [41]. Osteophytes were graded based on these identified stages of osteophyte development: in Stage I, mesenchymal condensates and evidence of chondrogenic differentiation are present; in Stage II, fibrocartilage develops with a mixture of cartilaginous and fibrous matrix components; in Stage III, the proliferating osteophyte shows a zonal organization similar to the fetal growth plate cartilage with extensive chondrocyte hypertrophy in the zones adjacent to ongoing endochondral bone formation; and in Stage IV, ‘mature’ osteophytes resemble largely articular hyaline cartilage. Lateral and medial regions of the tibial plateau and femoral condyles were each given a score of 0–4 for the four stages of osteophyte development. Scores from each site were averaged among the graders and summed for a total osteophyte score ranging from 0 to 16. Median scores for each experiment group were reported with quartile indicated.

### Immunohistochemistry for Collagen VI

Immunohistochemistry was performed to confirm the presence or absence of collagen VI in the articular cartilage of the knee. An IgG rabbit polyclonal anti-collagen VI antibody was used to bind a peptide near the amino terminus of the murine α1(VI) chain (sc-20649, Santa Cruz Biotechnology, CA). An Alexa Fluor® 488-conjugated secondary antibody, goat anti-rabbit IgG (Invitrogen/Molecular Probes, Carlsbad, CA) was used for primary antibody detection [27], [42]. Samples were further incubated with ethidium homodimer-1 (Molecular Probes, Carlsbad, CA) to stain nucleic acids. Samples were imaged using a 100×, 1.3-NA oil-immersion objective with differential interference contrast (DIC), an argon laser (excitation 488 nm), and a helium laser (excitation 633 nm). A band-pass filter (505–550 nm) and a long-pass filter (650 nm) collected emissions from the secondary antibody and the ethidium homodimer-1, respectively.

### Atomic Force Microscopy

For AFM measurements, the left mouse tibia was thawed, carefully dissected, and cut at mid-shaft with a razor blade to shorten its length. Hot-melt glue (Arrow Fastener Co., Saddle Brook, NJ) was used to secure the diaphysis of the tibia vertically within a small polystyrene Petri dish lid (Becton Dickenson, Franklin Lakes, NJ), leaving the tibial plateau near-horizontal within the lid. Phosphate-buffered saline (PBS, Gibco, Carlsbad, CA) at room temperature was added to cover the tibial plateau. The lid was secured to a glass microscope slide and oriented such that the anterior-posterior direction on the lateral tibial plateau was perpendicular to the AFM cantilever.

AFM cantilever tips were constructed by gluing (Norland optical adhesive #81, Norland Products Inc., Cranbury, NJ) 10-µm-diameter borosilicate microspheres (Duke Scientific Corporation, Palo Alto, CA) near the free end of a triangular silicon nitride AFM cantilever (Veeco, Santa Barbara, CA) with a nominal spring constant of 0.58 N/m [43]. Tips were coated with gold, ozone-cleaned, and functionalized with a monolayer of tri-ethylene glycol-terminated alkane thiol (SH-(CH_2_)_11_-EG_3_, Sigma Aldrich, St. Louis, MO) to inhibit tip fouling during the test procedure.

Friction was measured on the lateral tibial plateaus using a MFP-3D atomic force microscope (Asylum Research, Santa Barbara, CA) as described previously [43]. Briefly, friction was measured by tracking the lateral deflection signal as the probe scanned over the surface with a scan angle of 90. The area measured in each sample was grossly the midpoint of the lateral plateau, away from tendons, ligaments or meniscus. Normal force spring constants were found using the MFP-3D software provided by Asylum Research [44] and lateral calibration constants were calculated by the wedge method [45], [46]. For three different 50×50 µm cartilage areas, the probe was raster-scanned at 40 µm/s for 16 scan lines and 512 points captured per scan line. Each area was scanned at applied normal loads increasing from 20 to 100 nN [43]. The friction forces were averaged and graphed versus the applied normal load, after which the slope was taken as the coefficient of friction.

Roughness was calculated from an AFM image (128 scan lines by 128 points) of one of the friction sites on each tibia. The 50×50 µm field was scanned with a 20 nN applied normal load and a 100 µm/s scan speed. To avoid any plateau tilt effects on these data, a first-order (linear) flattening correction was applied and the root mean square (RMS) roughness recorded. The flattening technique corrects for any artifacts in image acquisition by fitting each scan line with a polynomial and subtracting it from the raw data.

AFM indentation was used to determine the elastic modulus of the cartilage surface via elastic tests previously described [47], [48], [49]. At each of the three 50×50 µm tibial plateau fields where friction was measured, 1 µm/s indentation testing was performed at 16 sites using a 4×4 layout. A sampling rate of 1 kHz and a force trigger of 100 nN was used. The elastic modulus was then calculated from the force vs. indentation data using a Hertz contact model for a hard sphere against an infinite plane in which the Poisson's ratio for the murine cartilage was assumed to be 0.20 [50], [51]. The moduli from these 16 sites were then averaged for each 50×50 µm field, leading to 3 separate values of indentation data for each limb evaluated.

### Scanning Microphotolysis (SCAMP)

SCAMP uses high laser power to simultaneously image and photobleach a single line segment that decreases in intensity over time as a function of the rate of photobleaching and rate of diffusion of the fluorescent molecule of interest. The diffusion coefficient and bleaching rate constant are determined by fitting a 3D theoretical diffusion-reaction model which accounts for the out-of-plane bleaching effects [52]. The left mouse femur was carefully dissected and soaked overnight in 70 kDa fluorescent dextran (25 mg/mL) at 4°C. A razor blade was used to slice sagittally through the lateral and medial condyles. The femur was placed in a coverslip chamber with the sliced condyle face positioned flush against the coverslip to allow a full-thickness view of the cartilage via the inverted microscope. All SCAMP experiments were performed in the extracellular matrix of the articular cartilage middle zone of any of the sliced, exposed faces [53].

As previously described [54], a 100×, 1.3-NA oil immersion lens at 6× zoom on a confocal laser scanning microscope (LSM 510, Zeiss) was used to bleach a line 1.44-µm wide (12 pixels) at a depth of 6 µm into the tissue. Bleaching was performed forward and backward for a total of 40 passes at two simultaneous excitation wavelengths, 458 and 488 nm. During bleaching, the emission intensity was also collected (long-pass filter, LP505) and stored as the image for that pass. At each site, five experiments were performed; their intensity values were averaged and median-filtered to minimize noise. For each test site, the experimental data was compared to simulated datasets using an unconstrained nonlinear optimization function; the resulting best fit established an appropriate value for the diffusion coefficient (*D*), which is the parameter of interest, and the space-dependent bleaching rate constant (*k*), which is a function of laser bleaching power and so varies with depth into the cartilage. All data processing, fits, and simulations were performed in Matlab (The Mathworks, Natick, MA). Diffusion coefficients were measured at 4–6 sites in each joint and the mean value was reported.

### Statistical Analyses

In Statistica (StatSoft, Tulsa, OK), each dataset was evaluated with the Shapiro-Wilks test for normal distribution; based on those outcomes, the non-normal elastic modulus data were log-transformed for statistical analysis. Next, multifactorial analysis of variance (ANOVA) was performed to assess significant (α = 0.05) main effects and interactive effects of genotype, sex, and age. Fisher's LSD post-hoc test was used to compare individual results where the ANOVA established a significant effect. A Kruskal-Wallis test of significance was performed on non-normal data after log-transformation.

## Results

**Figure 1:**
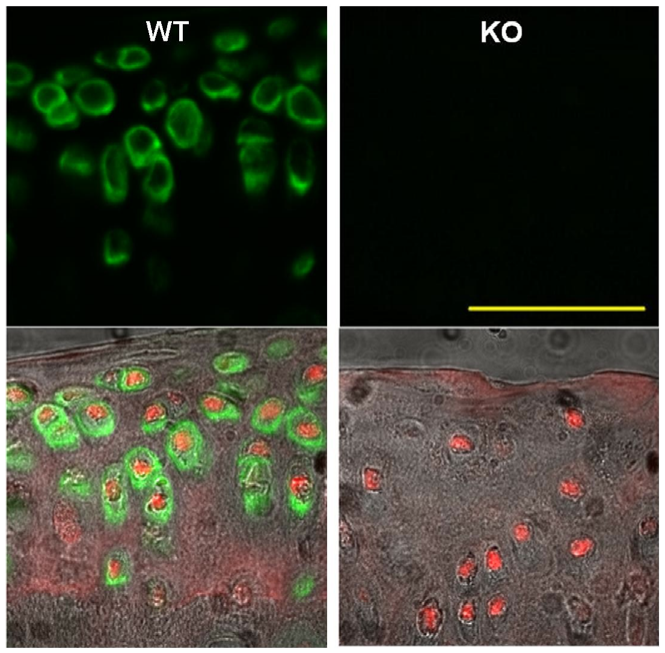
Immunolabeling for collagen VI in the articular cartilage of the mouse tibial plateau cartilage. Top row: Collagen VI is found exclusively in the pericellular region of chondrocytes in the wild-type PCM (left). Bottom row: Corresponding DIC images overlaid with collagen VI labeling (green) and nuclear staining (red). Cartilage of the *Col6a1^−/−^* mice (right) shows no labeling for collagen VI. Scale bar, 50 µm. WT = *Col6a1^+/+^*; KO = *Col6a1^−/−^*.

*Col6a1* ^−/−^ mice did not exhibit any major phenotypic abnormalities or show any increase in mortality at the time points examined. Immunohistochemistry staining confirmed that collagen VI was present in the PCM of *Col6a1^+/+^* cartilage and was absent from *Col6a1^−/−^* cartilage (Figure 1); in the *Col6a1^+/+^*tibia, the growth plate also showed significant labeling for collagen VI (*data not shown*).

### Tibial Epiphysis: Trabecular Bone

In the trabecular regions of the tibial epiphysis, the total volume, bone volume, bone volume fraction, bone tissue density, connectivity density, SMI, trabecular number, trabecular separation, and trabecular thickness were all measured by microCT (Table S1). The main effects of age (except total volume) and genotype (except bone tissue density) were significant. Briefly, morph metric values for *Col6a1^−/−^* mice were lower for bone volume, bone volume fraction, connectivity density, trabecular number, and trabecular thickness, but were higher for SMI and trabecular separation, as compared to *Col6a1^+/+^*.

**Figure 2:**
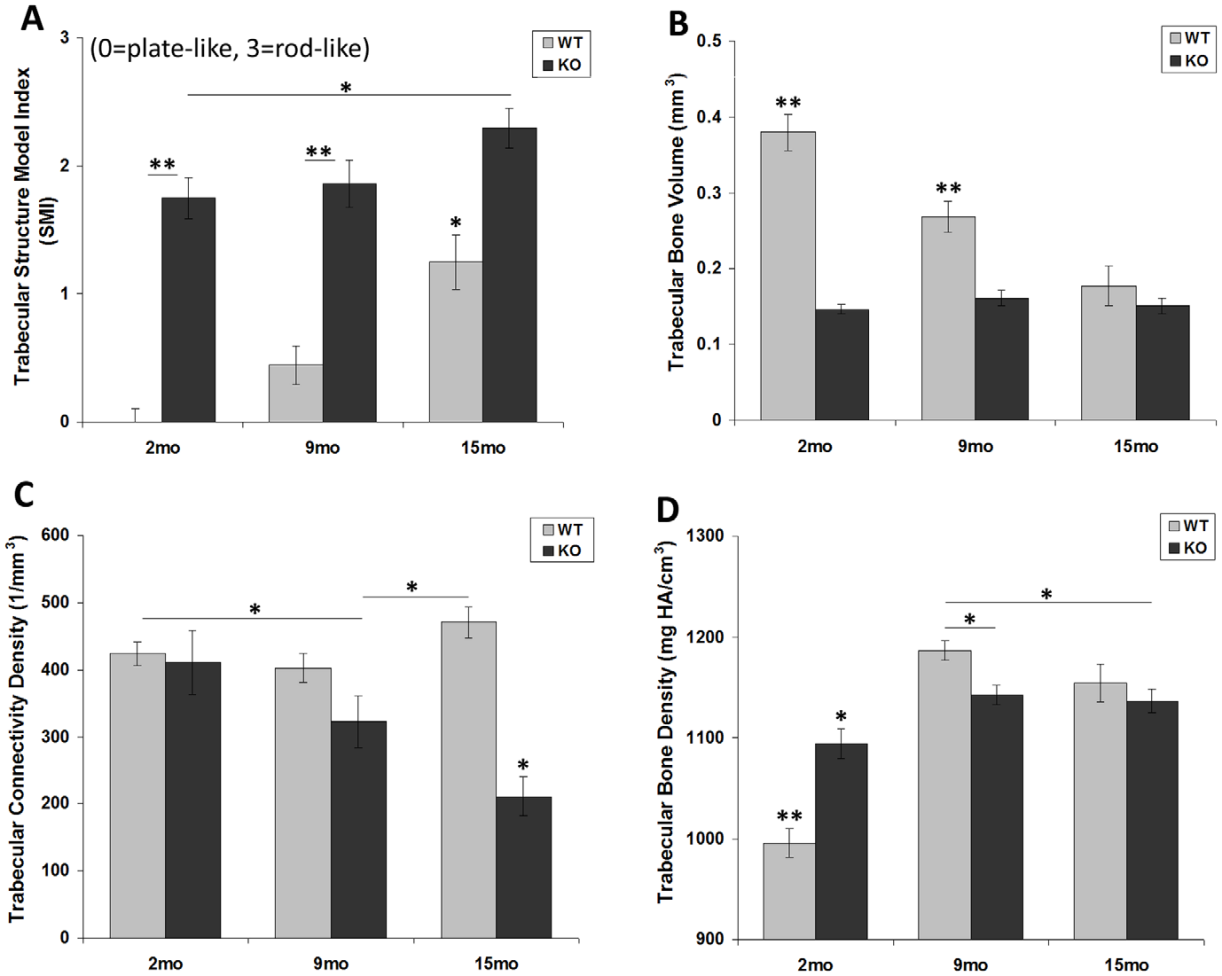
MicroCT measures of trabecular bone structure. In the tibial epiphysis of collagen VI null mice (*Col6a1^−/−^*), the structure model index (SMI, A) shows evidence of rod-like trabeculae from the earliest age, in contrast to the initially plate-like trabeculae of the wild-type mice. The trabecular bone volume (BV, B) is low from an early age and contributes to a higher bone tissue density (BD, D) in 2-month-old *Col6a1^−/−^* mice. With increasing age, the *Col6a1^−/−^* connectivity density (ConnDens, C) drops significantly at 15 months. Bars are mean ± SEM. Starred bars with no connecting lines are different from all other bars including each other. Horizontal lines connect statistically significantly different values. *p<0.05; **p<0.01. WT = *Col6a1^+/+^*; KO = *Col6a1^−/−^*.

**Figure 3:**
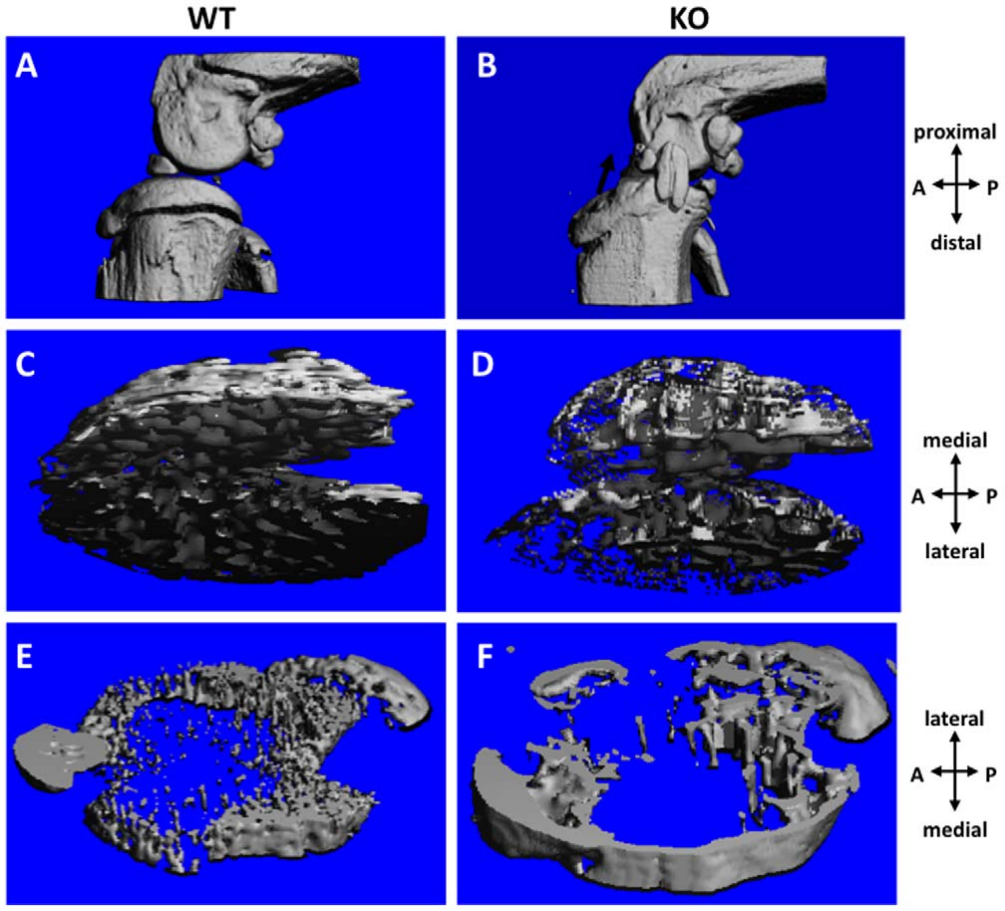
MicroCT images of 2-month-old mouse knees, showing the calcified structures of the right knees of a male wild-type mouse (left images: A, C, E) and a male *Col6a1^−/−^* mouse (right images: B, D, F). Top row (A, B) shows a medial view of the knee; note the ossified medial collateral ligament in the *Col6a1^−/−^* joint (arrow, B). Middle row (C, D) displays a distal view of the tibial trabecular bone, where the *Col6a1^−/−^* trabeculae are rod-like while the wild-type trabeculae are plate-like. Bottom row (E, F) shows a proximal view of the tibial metaphyseal region, where the wild-type bone is immature in comparison to the *Col6a1^−/−^* bone. WT = *Col6a1^+/+^*; KO = *Col6a1^−/−^*.

More specifically, the SMI of *Col6a1^−/−^* mice reflected “rod-like” structures (SMI closer to 3) from the youngest age through maturity, whereas the *Col6a1^+/+^*SMI indicated plate-like structures (SMI closer to 0) that became more rod-like over time (Figure 2A). Trabecular bone volume for *Col6a1^−/−^* mice remained smaller and constant, unlike the *Col6a1^+/+^* trabecular bone volume which dropped significantly with age (Figure 2B). In *Col6a1^−/−^* mice, total volume values increased with age to surpass the *Col6a1^+/+^* values (Table S1). The connectivity density dropped significantly with age in *Col6a1^−/−^* mice, whereas the *Col6a1^+/+^* connectivity density remained consistently higher (Figure 2C). With skeletal maturity (∼4 months old [55]), bone tissue density increased only 4% from 2 to 9 months in *Col6a1^−/−^* mice, while *Col6a1^+/+^* bone tissue density increased 19% (Figure 2D). MicroCT images comparing 2-month-old *Col6a1^+/+^* and *Col6a1^−/−^* trabecular bone are presented in Figure 3.

### Tibial Epiphysis: Cortical and Trabecular Bone

Several differences were observed in the total volume, bone volume, bone volume fraction, bone tissue density, and length of the bone in the tibial epiphysis. The main effects of age (except bone volume fraction) and genotype (except total volume and length) were significant (Table S1). Length and total volume showed increases with age that were similar for both genotypes. Bone volume increased steadily with age for *Col6a1^+/+^* while the bone volume in *Col6a1^−/−^* mice was lower at all time points as compared to *Col6a1^+/+^* and did not increase as drastically with age (46% vs. 23% respectively). The net result is that *Col6a1^−/−^* bone volume fraction remained unchanged while *Col6a1^+/+^* bone volume fraction increased (16%) between the 2 to 9 month time points, then leveled off. With skeletal maturity (∼4 months old [55]) *Col6a1^−/−^* bone tissue density increased only 5% versus 21% for *Col6a1^+/+^* measured between the 2 and 9 month time points.

### Tibial Metaphysis: Cortical and Trabecular Bone

**Figure 4:**
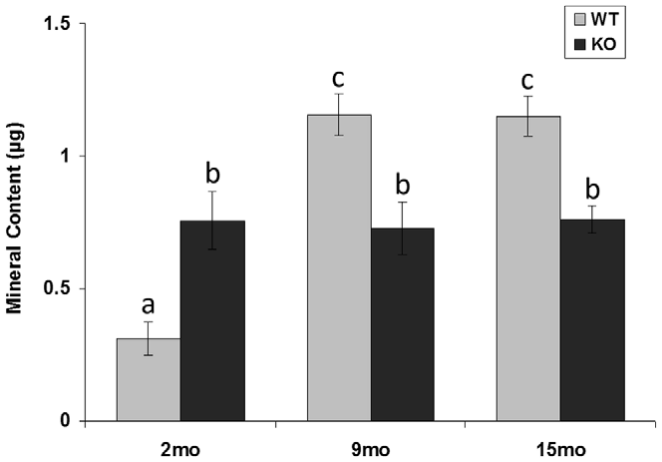
Mineral content of the *Col6a1^−/−^* tibial metaphysis. In the tibial metaphysis directly distal to the fibular attachment (25 microCT slices = 0.4 mm), wild-type mice quadrupled their mineral content from 2-months-old to 9-months-old. In contrast, collagen VI null mice already had an intermediate level of mineral content by 2 months of age; this mineral content never changed. Bars are mean ± SEM; those sharing the same letter are not statistically different; bars with different letters: *p*<0.01. WT = *Col6a1^+/+^*; KO = *Col6a1^−/−^*.

In the tibial metaphysis (Figures 3E and 3F), only bone volume and bone tissue density were quantified by microCT. Both showed a statistically significant main effect of age (but not of genotype) and a significant interactive effect of age-by-genotype (Table S1). Bone volume did not change significantly over time in *Col6a1^−/−^* mice, but *Col6a1^+/+^* bone volume increased 77% from 2 to 9 months. As with metaphyseal bone volume and epiphyseal bone density, metaphyseal bone density was influenced by skeletal maturity; *Col6a1^+/+^* bone tissue density increased 34% versus 7% in *Col6a1^−/−^* mice. Based on the mineral content calculated from the bone volume and bone tissue density values in this region, the *Col6a1^−/−^* mice did not gain any mineral content with age, whereas the mineral content increased nearly four-fold during skeletal maturation from 2 to 9 months in *Col6a1^+/+^* mice (Figure 4).

### Joint Capsule Thickness

**Figure 5:**
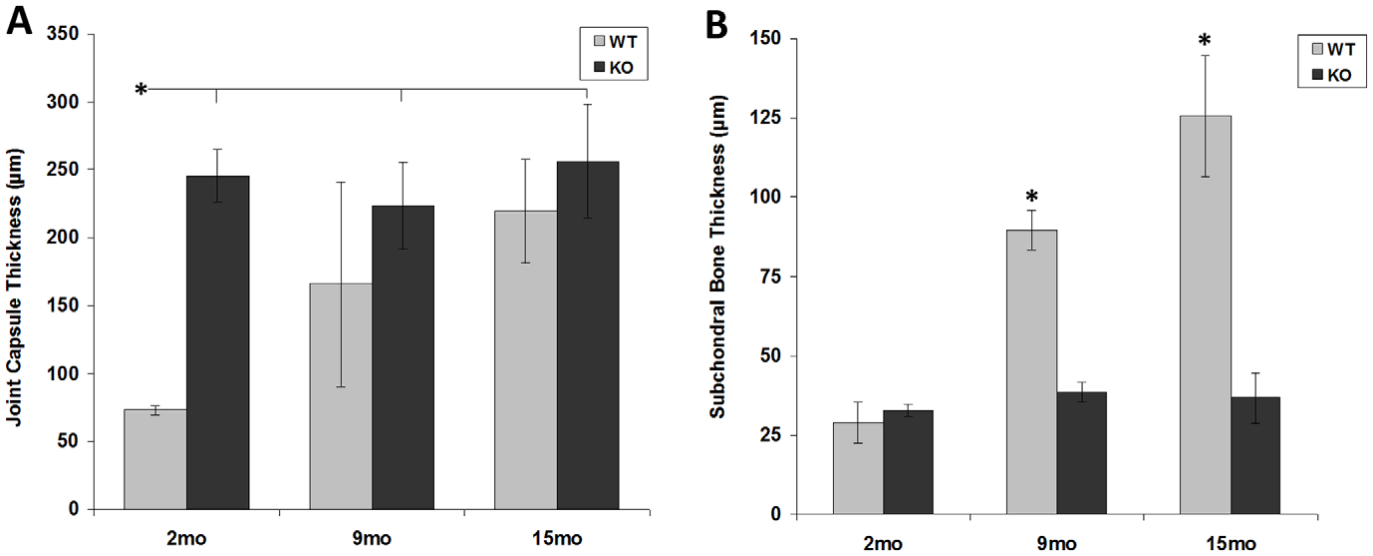
Effects of genotype on the joint capsule and subchondral bone. Joint capsule thickness on the medial side was high in *Col6a1^−/−^* mice from the youngest age but almost universally low in wild-type mice until the oldest age (A). Subchondral bone thickness, in contrast, shows that *Col6a1^−/−^* bone fails to thicken with age, unlike the wild-type bone (B). Bars are mean ± SEM. Starred bars with no connecting lines are different from all other bars including each other. Starred bar connected by notches on horizontal line to multiple bars is different from those values. *p<0.05, **p<0.01. WT = *Col6a1^+/+^*; KO = *Col6a1^−/−^*.

**Figure 6:**
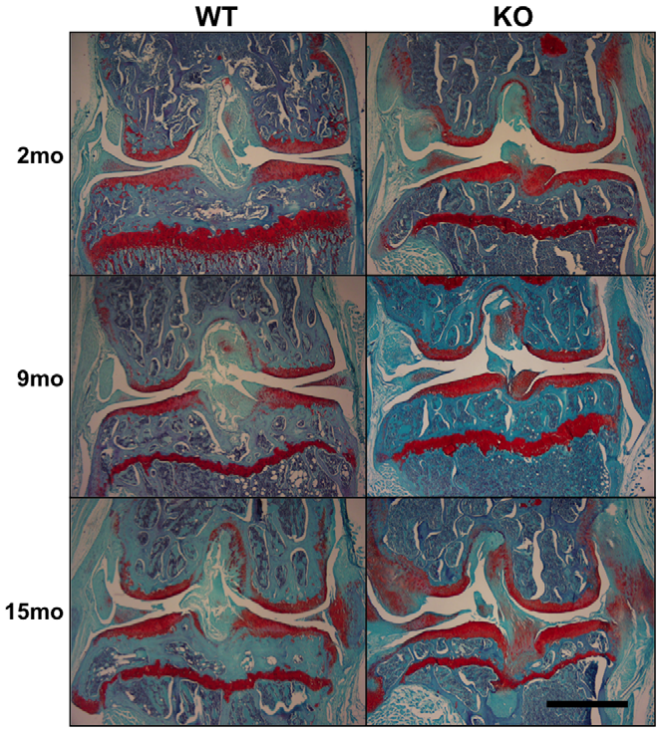
Histologic images revealed differences among wild-type and *Col6a1^−/−^* knees at 2, 9, and 15 months. Coronal tissue slices (7 µm thick) were stained with fast green for collagen (blue) and safranin-O for proteoglycans (proteoglycans), and Harris' hematoxylin for nuclei (black). The left side is lateral while the right side is medial. The collagen VI null knees (right column) include a thick and proteoglycan-rich medial collateral ligament, increased chondrophyte formation on the lateral femur, larger trabeculae in both bones, and at 2 months a more confined growth-plate staining relative to the wild-type knees (left column). Scale bar = 1 mm. WT = *Col6a1^+/+^*; KO = *Col6a1^−/−^*.

From the histology images, the joint capsule thickness (primarily the MCL) was measured adjacent to the medial meniscus and was affected by genotype (ANOVA, *p*<0.02) (Figure 5a). In the *Col6a1^−/−^* mice, the joint capsule was consistently thick at all ages, whereas the *Col6a1^+/+^* mice had initially thin joint capsules that thickened with age. Interestingly, most of the 9-month *Col6a1^+/+^* mice had a joint capsule thickness comparable to that of the 2-month *Col6a1^+/+^* mice (∼70 µm), yet the mean 9-month *Col6a1^+/+^* value was raised substantially by two very thick values (∼500 µm) in particularly degraded joints. Additionally, many of the *Col6a1^−/−^* joint capsules stained heavily for proteoglycan content (Figure 6), and a few had visible ossification.

### Subchondral Bone Thickness

From histology images, the mean subchondral bone thickness per joint was calculated by averaging the subchondral thickness at four sites: the lateral femoral condyle, the lateral tibial plateau, the medial femoral condyle, and the medial tibial plateau. The subchondral bone was thinner for the *Col6a1^−/−^* mice at all ages but thickened markedly with age for the *Col6a1^+/+^* mice (*p*<0.02 for each comparison) (Figures 5b). ANOVA confirmed the statistical significance (*p*<0.0002) of age, genotype, and age-by-genotype effects (Figure 5b).

The microCT measures of subchondral bone thickness confirmed these histology findings (Table S1). The microCT assessment of subchondral bone did result in numerically higher thickness measures than histologic assessment. However, the microCT measured only subchondral bone of the tibia, and some portion of calcified cartilage may be captured by the microCT thickness measures.

### Cartilage Degeneration

**Figure 7:**
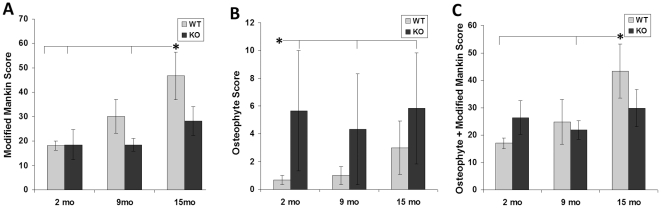
Effects of genotype on cartilage and joint structure. Cartilage degradation in *Col6a1^−/−^*mice did not significantly increase with age, whereas wild type cartilage increased from 2 to15 months. (A) Bars are mean ± SEM. Osteophyte score was initially low in *Col6a1^+/+^* mice and trended up with age, whereas severity of osteophytes began and remained high among the *Col6a1^−/−^* mice (B). Median with quartile bars shown. The combination of the two scores showed trends similar to that of the Modified Mankin score (C). Bars are mean ± SEM. Starred bar connected by notches on horizontal line to multiple bars is different from those values. *p<0.05, **p<0.01. WT = *Col6a1^+/+^*; KO = *Col6a1^−/−^*.

Using a modified Mankin score, the extent of cartilage degradation was determined in the lateral femoral condyle, lateral tibial plateau, medial femoral condyle, and medial tibial plateau of each joint. *Col6a1^−/−^* mice showed delayed or reduced cartilage degradation with age relative to the *Col6a1^+/+^* mice (Figure 7a). ANOVA revealed significant effects of side (medial or lateral, *p*<0.00003), but not bone (femur or tibia, *p* = 0.13), age (*p* = 0.06), genotype (*p* = 0.12) and age*genotype (*p* = 0.15) to be significant. In general, the cartilage showed an age-dependent increase in the osteoarthritic degeneration score; likewise, greater degradation was observed on the medial side of the joint as compared to the lateral side.

*Col6a1^−/−^* mice also showed a significant number of osteophytes (Figures 6). An osteophyte grading scheme was used to quantify the severity of these growths. As expected, *Col6a1^+/+^* mice showed age-dependent increases in osteophytes. *Col6a1^−/−^* mice showed higher osteophyte scores, starting at 2 months with a median score 8 times that of *Col6a1^+/+^* mice (5.7 vs 0.7, respectively) and remaining higher; although the effect decreased with age (Figure 7b). Nonparametric Kruskal-Wallis showed 2 month *Col6a1^+/+^* mice to be significantly different from *Col6a1^−/−^* mice at all time points.

The presence of osteophytes is generally not included in osteoarthritic grading schemes for mice as most mouse models of osteoarthritis do not show extensive osteophytes formation; however, in this case, osteophyte score was combined with the modified Mankin score to better assess the unique joint changes in these mice. This comprehensive score showed trends similar to that of the modified Mankin score; an age-dependent increase in the osteoarthritic degeneration score in *Col6a1^+/+^* mice and stable degeneration scores in *Col6a1^−/−^* mice (Figure 7c).

### Friction, Roughness, Elastic Modulus, and Diffusivity

The coefficient of friction of the articular cartilage of the tibial plateaus of the 2-month and 15-month mice ranged from 0.19 to 0.24. No statistically significant differences were observed in this property with time or genotype (Table S2).

RMS roughness was measured on tibial plateaus of all mice except one 15-month *Col6a1^−/−^* mouse, which was too rough to scan with this AFM setup. Age significantly influenced the roughness RMS (*p*<0.02), with the oldest mice having the roughest cartilage, but no differences with genotype were observed (Table S2).

Cartilage stiffness (elastic modulus) decreased significantly with age (*p*<0.02, Table S2). Cartilage from *Col6a1^−/−^* joints showed a trend for a lower elastic modulus at all age points but no statistically significant difference was observed (*p*<0.09).

The diffusivity of 70 kDa dextran through the ECM of femoral condyle cartilage averaged 13 µm^2^/s and was highly variable. Relative to the other values, diffusivity in the 2-month *Col6a1^−/−^* cartilage showed a trend for increased values: 17 µm^2^/s (*p* = 0.06 relative to 15-month *Col6a1^−/−^* data; *p*<0.05 relative to all other data) (Table S2).

## Discussion

The findings of this study provide new evidence of significant skeletal abnormalities associated with the lack of collagen VI. In particular, *Col6a1^−/−^* mice exhibited major differences in the trabecular bone within the proximal tibia, yet showed limited developmental changes in bone structure between 2 months and 9 months of age, as compared to the dramatic changes observed in *Col6a1^+/+^* mice. In contrast to the hip joint [27], *Col6a1^−/−^* mice showed delayed cartilage degeneration but drastically increased and earlier osteophyte development. Measures of the physical properties of the articular cartilage did not reveal consistent differences between genotypes. These findings suggest that alterations in bone structure are an early and important characteristic of *Col6a1^−/−^* mice, whereas the influence of collagen VI on the health and function of the synovial joint may depend significantly on the specific site.

An important new observation of this study is the significant influence that collagen VI may have on bone development and structure. Previous studies have shown delayed skeletal development and lower body bone mineral density in *Col6a1^−/−^* mice [27]. When compared to *Col6a1^+/+^*, the trabecular bone of *Col6a1^−/−^* mice showed lower bone volume, trabecular number, trabecular thickness, and connectivity density, but higher trabecular separation and SMI. Overall, these differences reflect trabecular bone structure with thinner, more widely spaced trabecular struts in *Col6a1^−/−^* mice, consistent with other mouse models bearing deletion or mutation of different collagen genes. For example, mice with the collagen I mutation *oim* present with fewer and thinner trabeculae in the femoral head relative to their wild-type counterparts [56]. *Col10a1^−/−^* mice show an early decrease in newly formed bony trabeculae and evidence of patchy mineralization of trabeculae, but an overall greater trabecular bone content in 4-week-old femurs [57]. Truncation of *Col2a1* in mice leads to lower trabecular bone volume fraction, lower trabecular thickness, and greater trabecular separation in the 3-month-old lumbar vertebral bodies [58]. Mice deficient in both *Col9a1^−/−^* and cartilage oligomeric matrix protein show a non-significantly lower trabecular mineral density at 1 month of age [59]. These previous studies, taken together with the findings of the current study, support the important role of various collagens in the assembly and maturation of bone.

The development and maturation of bone is highly dependent on mechanical loading, and a number of previous studies have shown changes in bone density and trabecular structure in association with altered muscle loads (e.g., [60]). Given the myopathy of *Col6a1^−/−^* mice [34], abnormal loading may be present at the knee and could influence trabecular bone characteristics. It is of interest to note that the differences between the *Col6a1^−/−^* and *Col6a1^+/+^* trabecular bone structures closely match the pattern of changes seen in studies of constrained locomotor modes [61], mechanically-altered loading (e.g., [62]), or disuse (e.g., [63]). Nonetheless, *Col6a1^−/−^* mice do not show altered activity levels (C57BL/6 background, [34]). Furthermore, our findings did not reveal characteristics of cartilage “disuse”, such as loss of proteoglycans from the cartilage matrix [64]. Further characterization of the biomechanical and behavioral characteristics of these mice may provide additional insights into the potential role of altered mechanical loads in relation to the bone changes observed in this study.

The mineral content of the tibial metaphysis was constant (∼0.75 µg HA) at all ages in the *Col6a1^−/−^* mouse. That value was intermediate to the *Col6a1^+/+^* mineral content, which was a low 0.31 µg at 2 months yet quadrupled with skeletal maturity to 1.15 µg at 9 and 15 months. Given previous evidence of skeletal changes during maturation in young *Col6a1^−/−^* mice, including abrupt whole-body bone mineral density (DXA) increases from 1-month old to 3-months old [27], it is surprising to see no indication of additional mineral deposition in the proximal tibial metaphysis as these mice age. However, abnormalities in collagen VI, a protein widespread in the bone growth plate, have been linked to a variety of ossification abnormalities including reduced bone mineral density [27], ligament ossification disorders [12], [13], [14], and delayed skeletal development [27]. These studies, together with the mineral deposition pattern observed, imply an important role for collagen VI in bone development.

Histologic analysis showed trends of progressive cartilaginous degeneration in the knees of CD1 *Col6a1^+/+^* mice, whereas the *Col6a1^−/−^* mice showed lower degeneration scores at all ages. Similarly, *Col6a1^−/−^* mice did not show thickening of the subchondral bone, a typical characteristic of osteoarthritis, whereas in the *Col6a1^+/+^* mice, the subchondral bone thickened more than 4-fold. However, *Col6a1^−/−^* mice showed significantly increased presence of osteophytes at the boundaries of the articular cartilage (Figure 6), which has been previously reported following local injection of transforming growth factor beta [65] or through cartilage-specific activation of beta catenin [66]. In contrast to this slower cartilage degradation pattern in *Col6a1^−/−^* knees, previous study of the hip joint identified accelerated and more severe degeneration for *Col6a1^−/−^* mice [27]. The disparity may be due to apparent site-specific effects of collagen VI mutations on connective tissue properties. For example, reduced collagen VI in Bethlem myopathy leads to proximal hypotonia and distal joint contractures [9], [70], whereas Ullrich congenital muscular dystrophy can be associated with hip laxity [10]. As joint laxity is a predisposing factor in the development of osteoarthritis (e.g., [71], [72]), differences in joint stability arising from muscle weakness and/or laxity may differentially influence specific joints of the body. Alternatively, the disparity in osteoarthritis progression between joints may be attributable to a potentially protective effect of large osteophytes that formed at the knee. It has been postulated that osteophytes act to stabilize the knee joint; in the knee, after a tear of the anterior cruciate ligament which acts to restrict sagittal movement of the tibia, osteophytes develop anteriorly and posteriorly and limit translocation of the femur on the tibia [67]. Additionally, the removal of osteophytes from human osteoarthritic knees has been shown to significantly increase versa-valgus instability [68], and in grade V disk degeneration of the spine, osteophyte formation decreases rotational movement in flexion and extension compared to lower grades, leading to spinal stabilization [69].

In previous studies, the loss of PCM stiffness has been associated with osteoarthritic changes in the joint. For example, human osteoarthritic cartilage exhibits reduced PCM stiffness as compared to non-osteoarthritic controls [73], [74]. Furthermore, *Col6a1^−/−^* and *Col6a1^+/−^* mice showed reduced PCM stiffness in the hip at 1 month of age, preceding any apparent changes in ECM histology or mechanical properties [27]. As the mechanical environment of the chondrocyte plays an important role in regulating its metabolic activity [75], [76], such alterations in PCM properties may ultimately contribute to chondrocyte dysfunction in osteoarthritis. For example, *Col9a1^−/−^* mice exhibit osteoarthritic changes and loss of cartilage mechanical properties in the medial compartment of the knee [77]. In the current study, cartilage extracellular matrix stiffness did not change between *Col6a1^−/−^* and *Col6a1^+/−^* mice, which is not surprising given that type VI collagen is only found in the pericellular matrix. We did not measure PCM mechanical properties in the knee, and thus it is not possible to determine if the lack of apparent cartilage degeneration in the knee was similarly associated with a lack of change in the PCM, despite the absence of type VI collagen. Thus future studies may wish to address this issue in a joint-specific manner using techniques such as AFM indentation to determine PCM properties [78], [79].

The measures of boundary friction coefficients were highly variable; on average, they showed no statistically significant change with age or genotype. The coefficients (0.21±0.14) corresponded well to previous friction data collected via this technique for 10-week-old (0.42±0.19) and 20-week-old (0.25±0.11) C57BL/6J mice [43], [48]. Elastic modulus values also varied widely, although *Col6a1^+/+^* mice generally had a higher modulus than *Col6a1^−/−^*. Overall, values were markedly lower than previously published values (10-week and 20-week C57BL/6J mice: ∼260 kPa and 354±158 kPa, respectively) [43], [48]. Finally, RMS roughness increased with age, with a trend toward rougher cartilage surface in the oldest mice. This trend is consistent with the progression of cartilage degradation with age, seen by histology.

The microscale diffusion technique used in this study provides a novel measure of macromolecular diffusivity of mouse cartilage. In general, the coefficients of diffusion measured in the mouse cartilage are similar to those obtained for cartilage of other species using this technique, i.e., ∼23 µm^2^/s in the extracellular matrix of porcine cartilage [53]. However, diffusion measurements revealed limited differences with age or genotype in this study. Average diffusive transport of 70 kDa dextran was fairly consistent at the older ages (9 and 15 months: 13 µm^2^/s), but at 2 months, the *Col6a1^−/−^* had a diffusivity 55% higher than that of the *Col6a1^+/+^* cartilage (11 µm^2^/s versus 17 µm^2^/s). This finding may reflect differences in the extracellular microstructure that may correspond to a developmental delay [27]. Because of the high cellularity of mouse cartilage at this age, the measurements may in fact represent differences in the PCM diffusivity due to the lack of collagen VI.

In conclusion, the findings of this study provide evidence of musculoskeletal abnormalities at the knee as a consequence of collagen VI absence. The trabecular bone characteristics reveal distinct structural differences between *Col6a1^−/−^* and *Col6a1^+/+^*bones. The proximal metaphysis mineral content did not increase with skeletal maturity in *Col6a1^−/−^*; the implications of this trend are unclear, but the cause may be altered mechanical loading due to joint laxity or muscle weakness, or potentially dysregulation of bone development in the absence of collagen VI. Of particular interest was the finding of delayed osteoarthritic degeneration in *Col6a1^−/−^* mice, although the physical properties of the knee cartilage were found to be similar to those of the *Col6a1^+/+^* mice. Clearly, further investigation of these parameters may provide new insights into the influence of the diverse characteristics of collagen VI loss or mutation on the musculoskeletal system.

## Supporting Information

Table S1 **MicroCT measurments of bone properties.** Bone properties were determined using microCT imaging and analysis as shown in Figure 3. Significant differences in bone properties were noted by genotype and age. Data are presented as mean ± standard deviation (n = 8 samples per group except n = 9 for wild-type 15 mo). *p<0.05, **p<0.01. The F-statistic is reported for p<0.5 in order of Age, Genotype, and Age-by-Genotype.
(XLSX)

Table S2 **Biomechanical and biophysical properties of articular cartilage.** The elastic modulus, roughness, and coefficient of friction were determined using atomic force microscopy, and the diffusion coefficient was determined using scanning microphotolysis. Data are presented as mean ± standard deviation, *p<0.05, **p<0.01. The F-statistic is included for significant findings.
(XLSX)
